# (1-Bromo­naphthalen-2-yl)acetonitrile

**DOI:** 10.1107/S1600536808017418

**Published:** 2008-06-19

**Authors:** Andria D. Harris, Amy D. Baucom, Jessica L. Brown, Daniel S. Jones, Craig A. Ogle

**Affiliations:** a Department of Chemistry, The University of North Carolina at Charlotte, 9201 University City Blvd, Charlotte, NC 28223, USA

## Abstract

The title compound, C_12_H_8_BrN, was prepared as a starting material for a Suzuki cross-coupling reaction with a pinacol ester. The torsion angle about the ring–methylene C—C bond is 30.7 (3)°, such that the N atom is displaced by 1.174 (4) Å from the plane of the naphthalene ring system.

## Related literature

A search of the Cambridge Structural Database [Version 5.29 (Allen, 2002 ▶); *CONQUEST* (Bruno *et al.*, 2002 ▶)] yielded one comparable structure, (4-bromo­naphthalen-2-yl)acetonitrile (Refcode BAGTEJ; Duthie *et al.*, 2001 ▶).
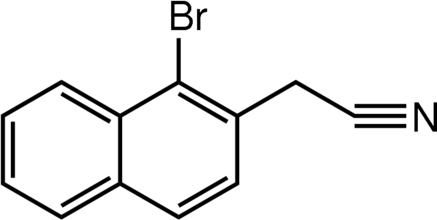


## Experimental

### 

#### Crystal data

C_12_H_8_BrN
*M* *_r_* = 246.1
Monoclinic, 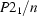

*a* = 11.3599 (13) Å
*b* = 7.2379 (8) Å
*c* = 11.8901 (15) Å
β = 102.538 (10)°
*V* = 954.31 (19) Å^3^
*Z* = 4
Cu *K*α radiation
μ = 5.47 mm^−1^
*T* = 295 (2) K
0.5 × 0.2 × 0.2 mm

#### Data collection

Enraf–Nonius CAD-4 diffractometer
Absorption correction: none
6502 measured reflections
1729 independent reflections
1558 reflections with *I* > 2σ(*I*)
*R*_int_ = 0.031
3 standard reflections every 75 reflections intensity decay: 2%

#### Refinement

*R*[*F* ^2^ > 2σ(*F* ^2^)] = 0.025
*wR*(*F* ^2^) = 0.063
*S* = 1.01
1729 reflections
127 parameters
H-atom parameters constrained
Δρ_max_ = 0.35 e Å^−3^
Δρ_min_ = −0.51 e Å^−3^

### 

Data collection: *CAD-4 EXPRESS* (Enraf–Nonius, 1994 ▶); cell refinement: *CAD-4 EXPRESS*; data reduction: *XCAD4* (Harms & Wocadlo, 1995 ▶); program(s) used to solve structure: *DIRDIF* (Beurskens *et al.*, 1999 ▶); program(s) used to refine structure: *SHELXL97* (Sheldrick, 2008 ▶); molecular graphics: *ORTEP-3 for Windows* (Farrugia, 1997 ▶); software used to prepare material for publication: *WinGX* (Farrugia, 1999 ▶).

## Supplementary Material

Crystal structure: contains datablocks global, I. DOI: 10.1107/S1600536808017418/fl2183sup1.cif

Structure factors: contains datablocks I. DOI: 10.1107/S1600536808017418/fl2183Isup2.hkl

Additional supplementary materials:  crystallographic information; 3D view; checkCIF report

## Figures and Tables

**Fig. 1.**
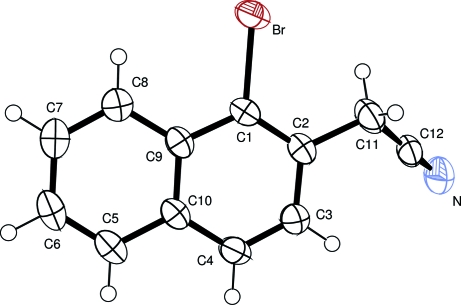
View of the title compound (IV) showing 50% probability displacement ellipsoids.

**Fig. 2.**
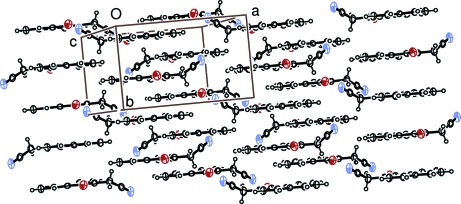
Diagram showing how the molecules of (IV) pack in alternating layers when viewed edge-on.

**Fig. 3.**
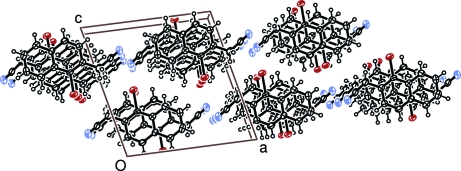
Diagram showing how the molecules of (IV) form columns when viewed along the b axis.

**Fig. 4.**
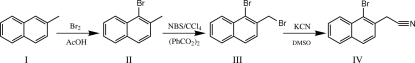
The formation of the title compound.

## supplementary crystallographic information

### Comment

The title compound (Fig.1) was prepared as a starting material for a Suzuki cross coupling reaction with a pinacol ester. The C11—C12—N angle is 178.4 (3)°, and the plane of that grouping makes an angle of 42.5 (1)° with the plane of the naphthalene ring, while the N atom is displaced 1.174 (4) Å from the plane of the naphthalene ring. As shown in Figs. 2 and 3, the molecules form alternating layers when viewed edge-on and form columns when viewed along the *b* axis.

A search of the Cambridge Structural Database [Version 5.29; (Allen, 2002); *CONQUEST*, Version 1.10 (Bruno *et al.*, 2002)] yielded one comparable structure, (4-bromonapthalen-2-yl)acetonitrile (Refcode BAGTEJ; Duthie *et al.*, 2001). In that structure the acetonitrile C—C—N angle was 179.3°, and the plane of that grouping made an angle of 23.1° with the plane of the naphthalene ring. The N atom was displaced 0.287 Å from the plane of the naphthalene ring.

### Experimental

Synthesis of 1-bromo-2-methylnaphthalene (II) (Fig. 4). A solution of 2-methylnaphthalene (I) in acetic acid was stirred while an equivalent amount of Br_2_ in acetic acid was added dropwise at a rate that allowed the bromine color to dissipate between drops. Upon completion of addition the mixture was allowed to stir for 1 h at which time the entire mixture was poured into water. The organic phase was separated and washed repeatedly with water to remove the acetic acid. The product was dried with K_2_CO_3_ and used in the next step without further purification.

Synthesis of 1-bromo-2-(bromomethyl)naphthalene (III). *N*-Bromosuccinimide (1 eq) and benzoylperoxide (0.01 eq) were added to a solution of (II) dissolved in CCl_4_. The reaction was then heated to reflux and the reaction progress was monitored with GC/MS. The reaction seemed to stall out at 3 h, and an additional portion of benzoylperoxide (0.01 eq) was added and allowed to reflux for an additional 3 h. The succinimide byproduct was removed by filtration from the cooled mixture. The CCl_4_ was removed and the product (III) was recrystallized from isooctane.

Synthesis of the title compound (IV). KCN (1.1 eq) was dissolved in DMSO with stirring. III (1.0 eq) was added along with additional DMSO to the stirred reaction mixture. A slight exotherm was observed, and the homogeneous mixture was allowed to stir overnight. The reaction was judged to be complete by GC/MS analysis. The reaction mixture was poured into water with stirring. The product precipitated upon addition to water. After filtering, the product was dried on a watch glass, and crystals for the diffraction study were obtained by recrystallization from a 2:1 mixture of 1,2-dimethoxyethane and ethanol.

### Refinement

H atoms were constrained using a riding model. The methylene C—H bond lengths were fixed at 0.97 Å, using an idealized tetrahedral geometry, with *U*_iso_(H) = 1.2 U_eq._ (C). The aromatic C—H bond lengths were fixed at 0.93 Å, with *U*_iso_(H) = 1.2 U_eq._ (C).

### Crystal data

**Table:**

C_12_H_8_Br_1_N_1_	*F*_000_ = 488
*M**_r_* = 246.1	*D*_x_ = 1.713 Mg m^−^^3^
Monoclinic, *P*2_1_/*n*	Cu *K*α radiation λ = 1.54184 Å
Hall symbol: -P 2yn	Cell parameters from 22 reflections
*a* = 11.3599 (13) Å	θ = 8.6–16.7º
*b* = 7.2379 (8) Å	µ = 5.47 mm^−^^1^
*c* = 11.8901 (15) Å	*T* = 295 (2) K
β = 102.538 (10)º	Prism, yellow
*V* = 954.31 (19) Å^3^	0.5 × 0.2 × 0.2 mm
*Z* = 4	

### Data collection

**Table:**

Enraf–Nonius CAD-4 diffractometer	θ_min_ = 4.9º
Nonprofiled θ/2θ scans	*h* = −13→13
Absorption correction: none	*k* = −8→8
6502 measured reflections	*l* = −14→14
1729 independent reflections	3 standard reflections
1558 reflections with *I* > 2σ(*I*)	every 75 reflections
*R*_int_ = 0.031	intensity decay: 2%
θ_max_ = 67.5º	

### Refinement

**Table:**

Refinement on *F*^2^	H-atom parameters constrained
Least-squares matrix: full	*w* = 1/[σ^2^(*F*_o_^2^) + (0.0276*P*)^2^ + 0.7384*P*] where *P* = (*F*_o_^2^ + 2*F*_c_^2^)/3
*R*[*F*^2^ > 2σ(*F*^2^)] = 0.025	(Δ/σ)_max_ < 0.001
*wR*(*F*^2^) = 0.063	Δρ_max_ = 0.35 e Å^−^^3^
*S* = 1.01	Δρ_min_ = −0.51 e Å^−^^3^
1729 reflections	Extinction correction: none
127 parameters	

### Special details

**Table:**

Geometry. All e.s.d.'s (except the e.s.d. in the dihedral angle between two l.s. planes) are estimated using the full covariance matrix. The cell e.s.d.'s are taken into account individually in the estimation of e.s.d.'s in distances, angles and torsion angles; correlations between e.s.d.'s in cell parameters are only used when they are defined by crystal symmetry. An approximate (isotropic) treatment of cell e.s.d.'s is used for estimating e.s.d.'s involving l.s. planes.

### Fractional atomic coordinates and isotropic or equivalent isotropic displacement parameters (Å^2^)

**Table:**

	*x*	*y*	*z*	*U*_iso_*/*U*_eq_	
Br	0.22261 (2)	0.12936 (4)	0.490726 (19)	0.04829 (11)	
N	−0.2239 (2)	−0.0251 (4)	0.2063 (2)	0.0621 (6)	
C2	0.0751 (2)	0.1301 (3)	0.2652 (2)	0.0364 (5)	
C9	0.29263 (19)	0.1292 (3)	0.2734 (2)	0.0339 (4)	
C1	0.1920 (2)	0.1289 (3)	0.32691 (18)	0.0334 (4)	
C10	0.2689 (2)	0.1334 (3)	0.1517 (2)	0.0353 (5)	
C4	0.1481 (2)	0.1373 (3)	0.0887 (2)	0.0416 (5)	
H4	0.132	0.1414	0.0087	0.05*	
C8	0.4149 (2)	0.1250 (3)	0.3346 (2)	0.0421 (5)	
H8	0.4327	0.1222	0.4147	0.051*	
C12	−0.1394 (2)	0.0450 (3)	0.2562 (2)	0.0434 (5)	
C11	−0.0312 (2)	0.1321 (4)	0.3232 (2)	0.0485 (6)	
H11A	−0.0082	0.0694	0.3968	0.058*	
H11B	−0.0498	0.2593	0.3384	0.058*	
C3	0.0552 (2)	0.1353 (3)	0.1439 (2)	0.0416 (5)	
H3	−0.0235	0.1373	0.1007	0.05*	
C5	0.3660 (2)	0.1332 (3)	0.0944 (2)	0.0435 (5)	
H5	0.3506	0.1351	0.0143	0.052*	
C7	0.5062 (2)	0.1252 (3)	0.2767 (2)	0.0488 (6)	
H7	0.5857	0.1219	0.3181	0.059*	
C6	0.4826 (2)	0.1302 (3)	0.1566 (2)	0.0484 (6)	
H6	0.5462	0.1314	0.1187	0.058*	

### Atomic displacement parameters (Å^2^)

**Table:**

	*U*^11^	*U*^22^	*U*^33^	*U*^12^	*U*^13^	*U*^23^
Br	0.05003 (18)	0.06614 (18)	0.03112 (16)	−0.00544 (11)	0.01412 (12)	−0.00341 (10)
N	0.0373 (12)	0.0871 (17)	0.0637 (15)	−0.0030 (12)	0.0149 (11)	−0.0019 (13)
C2	0.0366 (11)	0.0376 (10)	0.0387 (12)	−0.0030 (8)	0.0163 (10)	−0.0017 (9)
C9	0.0362 (11)	0.0297 (9)	0.0391 (11)	−0.0019 (8)	0.0152 (10)	−0.0016 (8)
C1	0.0398 (11)	0.0328 (10)	0.0302 (10)	−0.0025 (8)	0.0133 (9)	−0.0015 (8)
C10	0.0393 (11)	0.0323 (10)	0.0383 (12)	−0.0023 (8)	0.0170 (10)	0.0000 (8)
C4	0.0449 (13)	0.0510 (13)	0.0311 (11)	−0.0025 (10)	0.0132 (10)	0.0018 (9)
C8	0.0389 (12)	0.0459 (12)	0.0421 (13)	−0.0006 (9)	0.0099 (10)	0.0001 (10)
C12	0.0353 (12)	0.0531 (13)	0.0465 (13)	0.0060 (11)	0.0195 (11)	0.0062 (11)
C11	0.0406 (13)	0.0632 (15)	0.0476 (14)	−0.0070 (11)	0.0222 (12)	−0.0079 (11)
C3	0.0346 (11)	0.0530 (13)	0.0380 (12)	−0.0023 (10)	0.0092 (10)	0.0011 (10)
C5	0.0496 (14)	0.0430 (12)	0.0451 (13)	−0.0010 (10)	0.0260 (12)	0.0002 (10)
C7	0.0336 (12)	0.0531 (13)	0.0609 (16)	0.0003 (10)	0.0130 (12)	−0.0011 (11)
C6	0.0413 (13)	0.0485 (13)	0.0636 (17)	−0.0002 (10)	0.0292 (13)	0.0008 (11)

### Geometric parameters (Å, °)

**Table:**

Br—C1	1.903 (2)	C8—C7	1.364 (3)
N—C12	1.132 (3)	C8—H8	0.93
C2—C1	1.371 (3)	C12—C11	1.455 (4)
C2—C3	1.410 (3)	C11—H11A	0.97
C2—C11	1.515 (3)	C11—H11B	0.97
C9—C10	1.413 (3)	C3—H3	0.93
C9—C1	1.424 (3)	C5—C6	1.370 (4)
C9—C8	1.422 (3)	C5—H5	0.93
C10—C5	1.417 (3)	C7—C6	1.396 (4)
C10—C4	1.413 (3)	C7—H7	0.93
C4—C3	1.358 (3)	C6—H6	0.93
C4—H4	0.93		
			
C1—C2—C3	117.95 (19)	C12—C11—C2	114.1 (2)
C1—C2—C11	122.1 (2)	C12—C11—H11A	108.7
C3—C2—C11	119.9 (2)	C2—C11—H11A	108.7
C10—C9—C1	117.7 (2)	C12—C11—H11B	108.7
C10—C9—C8	118.25 (19)	C2—C11—H11B	108.7
C1—C9—C8	124.1 (2)	H11A—C11—H11B	107.6
C2—C1—C9	122.6 (2)	C4—C3—C2	121.7 (2)
C2—C1—Br	119.22 (15)	C4—C3—H3	119.1
C9—C1—Br	118.16 (17)	C2—C3—H3	119.1
C5—C10—C9	119.7 (2)	C6—C5—C10	120.2 (2)
C5—C10—C4	120.9 (2)	C6—C5—H5	119.9
C9—C10—C4	119.36 (19)	C10—C5—H5	119.9
C3—C4—C10	120.7 (2)	C8—C7—C6	121.2 (2)
C3—C4—H4	119.6	C8—C7—H7	119.4
C10—C4—H4	119.6	C6—C7—H7	119.4
C7—C8—C9	120.5 (2)	C5—C6—C7	120.1 (2)
C7—C8—H8	119.8	C5—C6—H6	120
C9—C8—H8	119.8	C7—C6—H6	120
N—C12—C11	178.4 (3)		
